# Impact of Systemic Pulmonary Shunt for Tetralogy of Fallot on Pulmonary Valve Annulus Growth and Midterm Pulmonary Valve Function

**DOI:** 10.1016/j.atssr.2026.02.025

**Published:** 2026-03-10

**Authors:** Tsutomu Hataoka, Fumiaki Shikata, Toru Okamura, Norihiko Oka, Takahiro Tomoyasu, Masahiro Kaneko, Yoshikiyo Matsunaga, Kenta Matsui, Sakura Horie, Kagami Miyaji

**Affiliations:** 1 Department of Cardiovascular Surgery, Kitasato University School of Medicine, Kanagawa, Japan; 2 Department of Cardiovascular Surgery, Gunma Children’s Medical Center, Gunma, Japan; 3 Department of Cardiac Surgery, The University of Tokyo Hospital, Tokyo, Japan; 4 Department of Cardiovascular Surgery, Jichi Children’s Medical Center Tochigi, Tochigi, Japan

## Abstract

**Background:**

Tetralogy of Fallot (TOF) often requires transannular patch repair, leading to pulmonary regurgitation. Systemic pulmonary shunt can be associated with pulmonary valve annulus (PVA) growth, thus potentially enabling valve-sparing repair. This study evaluated PVA growth after systemic pulmonary shunt and the midterm impact of valve-sparing repair on pulmonary valve function.

**Methods:**

We reviewed 54 patients with TOF who underwent staged repair with systemic pulmonary shunt. PVA *z* scores were compared before and after systemic pulmonary shunt. Freedom from pulmonary regurgitation (PR) was assessed. Freedom from PR was defined as PR less than moderate.

**Results:**

The median follow-up duration after intracardiac repair was 7.6 years (interquartile range [IQR], 3.1-10.8 years). In the staged repair group, the median PVA *z* score increased from −3.4 (IQR, −5.2 to −2.6) at the time of systemic pulmonary shunt to −2.7 (IQR, −4.4 to −1.8) before intracardiac repair. Freedom from PR at 8 years after intracardiac repair was higher in the valve-sparing repair group (84% vs 45%; *P* = .009). The pre–systemic pulmonary shunt PVA *z* score was a significant predictor of PVA growth (odds ratio, 1.87; *P* = .002), with a threshold of −4.1 predicting growth with 82.1% sensitivity and 72.7% specificity.

**Conclusions:**

PVA growth was observed in patients with TOF after systemic pulmonary shunt. The pre–systemic pulmonary shunt PVA *z* score predicts successful growth and informs surgical planning. Valve-sparing repair offers superior midterm freedom from PR compared with transannular patch repair.

**In Short**

▪ PVA growth was observed in patients with TOF after SPS.
▪ Pre-SPS PVA *z* score predicts growth, with −4.1 as a key threshold.
▪ VSR offers superior midterm freedom from PR compared with transannular patch repair.

Tetralogy of Fallot (TOF) repair often involves a transannular patch (TAP), leading to midterm pulmonary regurgitation (PR) and right ventricular dysfunction.^1^ Therefore, preserving the native pulmonary valve is a primary objective. Several institutions advocating an annulus-preserving approach report valve-sparing repair (VSR) rates of 36% to 80%.^2^ Systemic pulmonary shunts (SPSs) have been associated with pulmonary valve annulus (PVA) growth, with multiple studies demonstrating significant *z* score increases,^3^^,^^4^ thereby potentially enabling VSR in patients who otherwise would require TAP repair.^4^^,^^5^ However, factors contributing to successful post-SPS growth and long-term outcomes remain debated. This study evaluated PVA growth after SPS and the midterm impact of VSR on pulmonary valve function.

## Patients and Methods

This study was approved by the Institutional Review Board of Kitasato University School of Medicine in Kanagawa, Japan (approval number: B22-129).

From 2005 to 2023, 143 patients with TOF or double-outlet right ventricle with pulmonary stenosis (DORV/PS) underwent intracardiac repair (ICR) at our institution (Kitasato University School of Medicine, Kanagawa, Japan; Gunma Children’s Medical Center, Gunma, Japan; and Jichi Children’s Medical Center Tochigi, Tochigi, Japan). As a general policy, we do not perform primary ICR in the neonatal period for standard TOF. Our standard management for asymptomatic or stable patients is primary repair at 3 to 6 months of age. Among these 143 patients, 54 patients who underwent staged repair met the inclusion criteria for this study. The sole inclusion criterion was requiring a staged repair initiated with an SPS. Indications for this palliative strategy included cyanotic spells, symptomatic hypoxemia preventing discharge, low body weight, or significant pulmonary artery hypoplasia (Supplemental Table).

Patient characteristics are summarized in Table 1. Sixteen patients underwent VSR, and 38 required TAP repair. All 54 patients underwent an SPS procedure as the initial step, followed by ICR at a subsequent stage. VSR was considered when the PVA *z* score exceeded −3.0. Clinical, operative, and echocardiographic data were retrospectively reviewed. Outcomes included PVA growth (measured as *z* score) before and after SPS, freedom from PR, right ventricular outflow tract (RVOT) stenosis, and reintervention rates after ICR.

**Table 1:** Patients’ Demographic Characteristics

Characteristics	Total (n = 143)	Primary Repair (n = 89)	Staged Repair (n = 54)	*P* Value
Male/female	85/58	53/36	32/22	.97
VSR/TAP	53/90	37/52	16/38	.15
ICR, mos	8.0 (5.6-13.7)	7.7 (5.4-11.8)	9.3 (5.0-14.7)	.11
Body weight at ICR, kg	7.3 (6.4-8.4)	7.3 (6.4-8.4)	7.0 (6.4-8.4)	.77
Body weight at birth, kg	2.8 (2.5-3.1)	2.8 (2.3-3.2)	2.8 (2.6-3.1)	.30
Follow-up, y	7.6 (3.1-10.8)	7.6 (3.6-10.8)	7.4 (3.0-11.1)	.67
Operative mortality	0	0	0	…
Late mortality	1	1	0	…

Values are n, median (interquartile range), or *P*.

ICR, intracardiac repair; TAP, transannular patch; VSR, valve-sparing repair.

Reintervention was defined as surgical or catheter intervention on the RVOT or pulmonary arteries after ICR. Freedom from PR was defined as PR less than moderate. Continuous variables were expressed as median with interquartile range (IQR) and were compared using the Mann-Whitney *U* test. Categorical variables were compared using the Fishers exact test. Kaplan-Meier survival analysis was used to evaluate freedom from adverse events.

Multivariable logistic regression analysis was performed to identify predictors of PVA growth, defined by greater than −3.0 of the PVA *z* score before ICR. The variables included in the multivariable logistic regression analysis are listed in Table 2 The interval from SPS to ICR was evaluated but was excluded from the final multivariable model because of its significant correlation with the pre-SPS PVA *z* score. In contrast, the RVOT size indexed to body surface area showed no significant correlation with the pre-SPS PVA *z* score and was therefore retained in the model. A *P* value <.05 was considered statistically significant. The statistical analyses were performed using JMP Pro software version 18.0 (SAS Institute).

**Table 2:** Predictive Values of Pulmonary Valve Annulus Growth Achievement After Systemic Pulmonary Shunt

Variables	Univariable	Multivariable		
	*P* Value	Odds Ratio	95% CI	*P* Value
RVOT size/BSA (mm/mm^2^)	.02	0.87	0.74-0.99	.03
VSD size/BSA (mm/mm^2^)	.09	…	…	…
Graft size/BSA	1.00	…	…	…
Interval from SPS to ICR days	.06	…	…	…
Preoperative PV annulus *z* score	.0004	1.87	1.21-3.55	.002

BSA, body surface area; ICR, intracardiac repair; PV, pulmonary valve; RVOT, right ventricular outflow tract; SPS, systemic pulmonary shunt; VSD, ventricular septal defect.

## Results

The demographic characteristics are shown in Table 1. Of 143 total patients, 89 underwent primary ICR, including 23 patients with DORV/PS. A total of 55 patients underwent SPS; 1 patient (1.8%) with a ring chromosome 18 abnormality died of complications of complete atrioventricular block 80 days after SPS, and shunt patency was confirmed. The remaining 54 patients successfully proceeded to ICR and constitute the cohort for this analysis. In these 54 patients, including 23 with DORV/PS, there was no operative or late mortality. Patients underwent SPS at a median age of 44 days (IQR, 20-101 days) and at a median weight of 3.8 kg (IQR, 3.2-4.9 kg). Nineteen patients (35.2%) were neonates.

Comparisons of baseline variables between the VSR (n = 16) and TAP (n = 38) groups are shown in Table 3. Among the 54 patients, 16 underwent VSR and 38 had TAP repair, including 9 and 14 patients with DORV/PS, respectively. The median time to ICR was 9.3 months (IQR, 5.0-14.7 months).The median follow-up duration after ICR was 7.6 years (IQR, 3.1-10.8 years). In the staged repair group, the median PVA *z* score increased from −3.4 (IQR, −5.2 to −2.6) at the time of SPS to −2.7 (IQR, −4.4 to −1.8) before ICR. Additionally, the median PVA *z* score in the primary repair group changed from −1.9 (IQR, −2.3 to −1.2) at birth to −2.2 (IQR −3.3 to −1.3) before ICR.

**Table 3:** Baseline and Preoperative Variables of Patients Undergoing Systemic Pulmonary Shunt

Variables	VSR (n = 16)	TAP (n = 38)	*P* Values
At the time of SPS			
Age, d	38 (19-80)	46 (21-103)	.46
Body weight, kg	3.8 (3.3-4.7)	3.8 (3.1-5.1)	.57
PVA *z* score	−3.2 (−4.4 to −2.8)	−4.1 (−5.1 to −2.7)	.35
Using 3.5-mm graft, n	7/16 (43.8)	19/38 (50)	.61
At the time of ICR			
Interval from SPS to ICR, d	219 (149-316)	237 (148-348)	.45
Body weight, kg	6.8 (6.1-8.2)	7.4 (6.4-8.6)	.21
PVA *z* score	−1.9 (−3.4 to −1.3)	−3.1 (−4.9 to −2.1)	.03

Values are median (interquartile range), n/N (%), or *P*.

ICR, intracardiac repair; PVA, pulmonary valve annulus; SPS, systemic pulmonary shunt; TAP, transannular patch; VSR, valve-sparing repair.

Freedom from PR after ICR was significantly higher in the VSR group compared with the TAP group (84% vs 45% at 8 years after ICR) (*P* = .009) (Figure A). Freedom from RVOT stenosis (84% vs 93%; *P* = .73) and freedom from reintervention (65% vs 65%; *P* = .93) were comparable. Multivariable analysis identified a pre-SPS PVA *z* score as the significant predictor of successful PVA growth after SPS (odds ratio, 1.87; 95% CI, 1.21-3.55; *P* = .002) (Table 2). Receiver operating characteristic analysis identified a cutoff value of −4.1 (area under the curve, 0.72; sensitivity, 82.1%; specificity, 72.7%) (Figure B).

**Figure.**
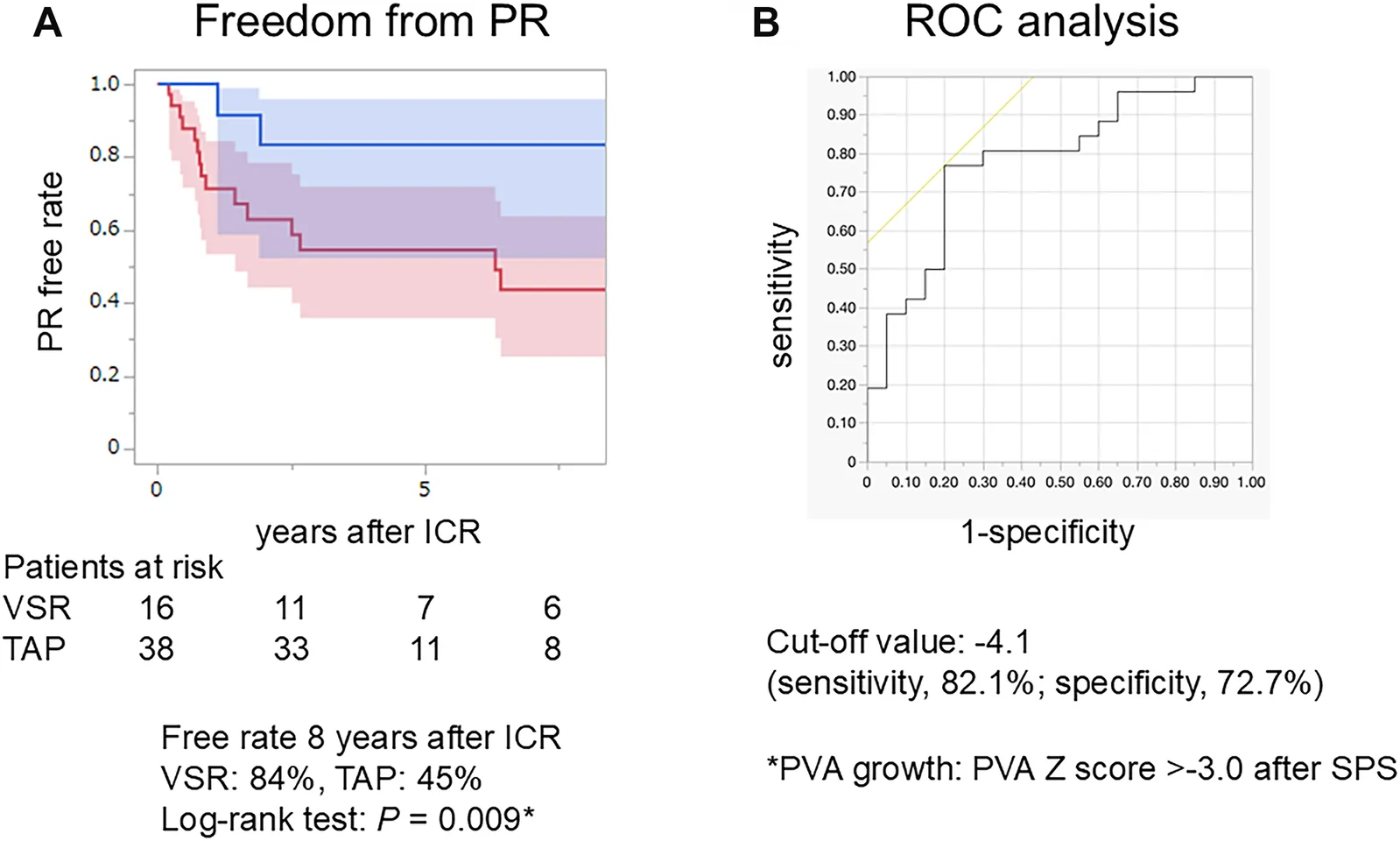
(A) Freedom from pulmonary regurgitation (PR) after intracardiac repair. The valve-sparing repair (VSR) group (blue line) shows significantly higher freedom from PR compared with the transannular patch (TAP) group (red line). (B) Receiver operating characteristic (ROC) curve for pre–systemic pulmonary shunt (SPS) pulmonary valve annulus (PVA) *z* score predicting successful PVA growth. Area under the curve = 0.72. (ICR, intracardiac repair.)

## Comment

In patients with TOF and hypoplastic PVA, SPS has been reported to be associated with PVA growth. If SPS allows for enhanced PVA growth, this strategy may result in later valve-sparing options for patients who otherwise may have required early TAP repair.^4^, ^5^, ^6^ Nakashima and colleagues^4^ reported that the increased left ventricular volume by Blalock-Taussig shunt may increase pulmonary blood flow through the pulmonary valve from the ventricular septal defect. However, the factors contributing to the PVA’s successful growth after SPS and the long-term outcomes are still debated.

We observed significant PVA growth in the VSR group; however, this was not universal. Multivariable analysis identified the pre-SPS PVA *z* score as a significant predictor, with a receiver operating characteristic–derived cutoff value of −4.1. Patients with PVA *z* scores lower than this threshold were significantly less likely to achieve sufficient PVA growth for VSR. That underscores the importance of careful preoperative assessment of PVA size. Patients with a PVA *z* score smaller than −4.1 may benefit from an early discussion about alternative surgical options such as RVOT stenting or early primary TAP repair. We also observed that a larger RVOT size indexed to body surface area was associated with limited PVA growth.

Although no statistically significant correlation was found between the RVOT index and the pre-SPS PVA *z* score in our cohort, this finding may suggest a phenomenon related to growth potential. Patients with a larger RVOT index may have a relatively preserved PVA initially, leaving less room for significant growth compared with patients with severe hypoplasia who undergo successful palliation.

It is widely accepted that achieving VSR is a primary goal in the surgical management of TOF because it is associated with superior long-term outcomes compared with TAP repair, provided there is no significant residual right ventricular pressure load.^7^ Blais and colleagues^8^ reported that patients who underwent a VSR had increased 30-year survival, fewer cardiovascular reinterventions, and fewer pulmonary valve replacements compared with individuals who underwent TAP repair, even in the presence of significant residual pulmonary stenosis.

Whereas freedom from PR was significantly higher in the VSR group, freedom from RVOT stenosis did not differ between groups. This finding is consistent with existing evidence suggesting that although TAP repair is effective in relieving RVOT stenosis, it is associated with significant midterm PR leading to right ventricular dilation, heart failure, and the need for pulmonary valve replacement. Given the substantial difference in PR rates between VSR and TAP repair, achieving VSR whenever possible should be a priority in surgical planning because it offers superior long-term outcomes.

Freedom from reintervention was similar between the groups, with both VSR and TAP repair showing a 65% freedom rate from reintervention at 8 years. This relatively low rate reflects our institutional proactive management philosophy with a low threshold for catheter-based intervention to optimize pulmonary artery architecture, rather than surgical failure.

Limitations of our study include its retrospective design and the inherent selection bias of a staged-repair cohort with severe anatomic features and the variability of echocardiographic measurements in neonates. Additionally, regarding the factors influencing PVA growth, we excluded the interval from SPS to ICR from the multivariable model for statistical reasons. However, we acknowledge that this interval is not determined solely by the initial PVA size; factors such as shunt reinterventions or branch pulmonary artery characteristics may also allow for a longer duration of palliation relative to hypoxia. Excluding this variable implies that the complex interplay between the duration of flow and growth potential was not fully adjusted for.

In conclusion, this study observed subsequent PVA growth in patients with TOF after SPS that may be associated with VSR in approximately 30% of patients. However, data from large multiinstitutional databases, such as The Society of Thoracic Surgeons Congenital Heart Surgery Database, indicate that an initial palliative shunt does not necessarily reduce TAP repair rates.^9^ The discrepancy between these results and our experience may reflect differences in patient selection criteria and institutional surgical strategies.

It is important to interpret the predictive value of the pre-SPS PVA *z* score with caution. A postgrowth *z* score of −3.0 is still considered a borderline dimension for VSR. Therefore, the cutoff value of −4.1 identified in our analysis should not be regarded as a definitive threshold for VSR success. We demonstrate that even with this high-risk subgroup, VSR was ultimately achievable in 30% of patients.

We present a practical, preoperative prognostic tool (the pre-SPS PVA *z* score) that may help predict which of these high-risk patients are most likely to achieve the desired goal of ultimate pulmonary valve preservation. Ultimately, the choice of surgical strategy must always rely on direct intraoperative assessment of the PVA. Although staged repair entails cumulative risks, including 2 sternotomies and potential interstage mortality, an annual report by the Japanese Association for Thoracic Surgery demonstrated low mortality for both shunts and TOF repair.^10^ We believe that the potential midterm benefit of pulmonary valve preservation justifies this strategy in selected high-risk patients.

## Declaration of Generative AI and AI-Assisted Technologies in The Writing Process

During the preparation of this work the authors used ChatGPT in order to improve the clarity and readability. After using this tool/service, the authors reviewed and edited the content as needed and take full responsibility for the content of the publication.
