# Supplementary material for: Impact of Systemic Pulmonary Shunt for Tetralogy of Fallot on Pulmonary Valve Annulus Growth and Midterm Pulmonary Valve Function
Source: Ann Thorac Surg Short Rep. 2026 Mar 10;4(3):857–62. doi: 10.1016/j.atssr.2026.02.025 (PMC13555404; doi:10.1016/j.atssr.2026.02.025)
Supplement: Supplementary file 1 [file mmc1.docx]

Supplementary table

The Indication of SPS

|  | Cyanotic spells | Hypoxemia  preventing discharge | Low body weight | Pulmonary artery  hypoplasia |
| --- | --- | --- | --- | --- |
| The number of cases | 37 | 10 | 1 | 6 |
